# Supplementary material for: The genome sequence of the Crescent Groundling, Teleiodes luculella (Hübner, 1813)
Source: Wellcome Open Res. 2024 Mar 11;9:143. [Version 1] doi: 10.12688/wellcomeopenres.21090.1 (PMC11319900; doi:10.12688/wellcomeopenres.21090.1)
Supplement: Peer review history

# Reviewer report: Reviewer response for version 1

**DOI:** 10.21956/wellcomeopenres.23330.r90454

**Published:** 2024-08-12

**Authors:** Axel Künstner

**Affiliations:** 1 University of Lübeck, Lübeck, Germany

The manuscript presents the genome assembly of the Crescent Groundling, *Teleiodes luculella*. The manuscript exhibits clear writing and sound methodologies. The raw data for this study was submitted to ENA.

 Besides one minor comment (see below), I have no further complaints.

**Minor comment:**

 In the Background section, there is the following unassociated sentence: 'Click or tap here to enter text'.

Are sufficient details of methods and materials provided to allow replication by others?

Yes

Is the rationale for creating the dataset(s) clearly described?

Yes

Are the datasets clearly presented in a useable and accessible format?

Yes

Are the protocols appropriate and is the work technically sound?

Yes

Reviewer Expertise:

Bioinformatics, Cancer research, Microbiome

I confirm that I have read this submission and believe that I have an appropriate level of expertise to confirm that it is of an acceptable scientific standard.

# Reviewer report: Reviewer response for version 1

**DOI:** 10.21956/wellcomeopenres.23330.r90450

**Published:** 2024-08-07

**Authors:** Wai Lok So

**Affiliations:** 1 School of Life Sciences, The Chinese University of Hong Kong, Hong Kong, Hong Kong

The authors sequenced the Crescent Groundling, *Teleiodes luculella, *yielding a high-quality chromosomal-level genome, together with the sex-chromosome and the mitochondrial genome. The methods are standard, sound, and logical. Sufficient details have been given regarding sample preparation, genome sequencing, and assembly. The genome and other associated materials have been properly deposited, which is accessible and reusable for future users.

 Though the genome sequencing part is well done, I would like to see a bit more background on this moth species, especially its special biology or ecological significance. The inclusion of additional biological details of this species in the "Background" would be greatly appreciated.

Are sufficient details of methods and materials provided to allow replication by others?

Yes

Is the rationale for creating the dataset(s) clearly described?

Yes

Are the datasets clearly presented in a useable and accessible format?

Yes

Are the protocols appropriate and is the work technically sound?

Yes

Reviewer Expertise:

myriapod biology, invertebrate biology, evolution, genomics, molecular biology, soil biodiversity, invertebrate endocrinology

I confirm that I have read this submission and believe that I have an appropriate level of expertise to confirm that it is of an acceptable scientific standard.
